# Kounis Syndrome Triggered by Medications and an Illicit Substance: A Report of a Rare Case

**DOI:** 10.7759/cureus.83608

**Published:** 2025-05-06

**Authors:** Saif M Srouji, Yazan Haroun, Sravan Vemuri, Muhammad U Bakhsh

**Affiliations:** 1 Internal Medicine, Saint Agnes Medical Center, Fresno, USA; 2 Cardiology, Saint Agnes Medical Center, Fresno, USA

**Keywords:** acs (acute coronary syndrome), allergic mi, cardiovascular disease, electrocardiography (ecg), epinephrine adverse effects, internal medicine-cardiology, kounis case study, kounis syndrome (ks), st-elevation myocardial infarction (stemi), systemic allergic conditions

## Abstract

Kounis syndrome (KS) is an underrecognized and critical condition of acute coronary syndrome (ACS) triggered by an allergic reaction, via histamine-mediated coronary vasospasm and plaque disruption. We present a case of type 1 KS in a 42-year-old male patient who developed ST elevation myocardial infarction following an allergic reaction to epinephrine, diphenhydramine, and cocaine. Initially stable, he later experienced worsening chest pain with a significant rise in troponin. Coronary angiography with intravascular ultrasound (IVUS) imaging revealed a layered left anterior descending (LAD) thrombus with possible acute plaque rupture but no significant coronary artery disease (CAD) otherwise, which was successfully managed with medical therapy. This case underscores the importance of early recognition, cardiology involvement, and balancing treatment geared toward allergic and cardiac manifestations. This report aims to raise awareness of KS, and its documented exposures are essential for timely diagnosis, targeted management, and improved patient outcomes, as there would be more focus on treating the allergic reaction in these cases, which might be overlooked if ACS was to be attributed to CAD as the reason.

## Introduction

Kounis syndrome (KS) was first described by Kounis and Zavras in 1991 as a clinical entity characterized by ischemic cardiac manifestations occurring in the context of an allergic, anaphylactic, or anaphylactoid reaction [1]. Based on its clinical presentation, KS has been classified into three types: vasospastic allergic angina (type 1), allergic myocardial infarction (type 2), and stent thrombosis (type 3) [2]. The syndrome has been reported in association with various allergens, including drugs, most commonly nonsteroidal anti-inflammatory drugs (NSAIDs) and antibiotics, foods, and environmental exposures [3,4]. While initially considered rare, KS is now recognized as an underdiagnosed condition, with increasing reports highlighting its occurrence across diverse populations, spanning different ethnicities, age groups (ranging from two to 90 years), and geographic regions [5]. It is estimated that its prevalence in the United States is a mere 1% among patients hospitalized for allergic reactions [6].

Here, we present a case of KS that is more consistent with vasospastic allergic angina or type 1 KS, as is suspected after the use of cocaine and later the administration of epinephrine, both strong mediators for vasospasm. It is of special interest, as multiple substances were identified as culprits in addition to the interesting finding of a layered thrombus on angiography and intravascular ultrasound (IVUS). Management of this condition was done medically, as no significant obstructive coronary artery disease (CAD) was found, and the patient recovered before finally being discharged home.

## Case presentation

**Figure 1:**
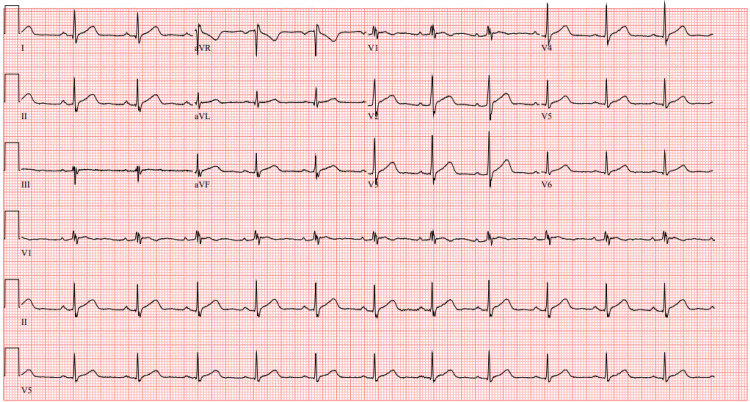
Initial ECG Initial ECG taken in the ED showing normal sinus rhythm at a heart rate of 70 bpm and no acute ischemic changes ECG: electrocardiogram; ED: emergency department

A 42-year-old Hispanic male patient with a past medical history of asthma presented to the emergency department (ED) complaining of shortness of breath and a generalized skin rash that started 30 minutes prior to his presentation after eating cabbage a few hours prior. He noted that he had not had any previous allergic reactions after eating cabbage and that this kind of complaint is new. En route to the hospital, he had been given diphenhydramine 50 mg intramuscularly (IM). Upon evaluation in the ED, his vital signs were as follows: heart rate (HR) of 76 bpm, blood pressure (BP) of 115/79 mmHg, respiratory rate (RR) of 17 breaths/min, SpO_2_ of 98%, and temperature of 36.6°C. Physical exam revealed a well-built young gentleman, who did not seem to be in respiratory distress. An urticarial rash was noticed across his chest and upper extremities. Upon examination of his respiratory system, it was noted that the oropharynx was patent without swelling, and his lungs were clear bilaterally with good air entry and without wheezing. He received dexamethasone 10 mg intravenously (IV), diphenhydramine 25 mg IV, famotidine 20 mg IV, and ibuprofen 600 mg orally (p.o.) for anti-inflammatory activity, in addition to a 1 L bolus of normal saline. Approximately 90 minutes later and while still being observed in the ED, he started complaining of sudden-onset chest pain. An initial electrocardiogram (ECG) (Figure 1) was unremarkable and showed normal sinus rhythm at a HR of 70 bpm without associated acute ischemic changes. No other actions were taken at this time.

**Figure 2:**
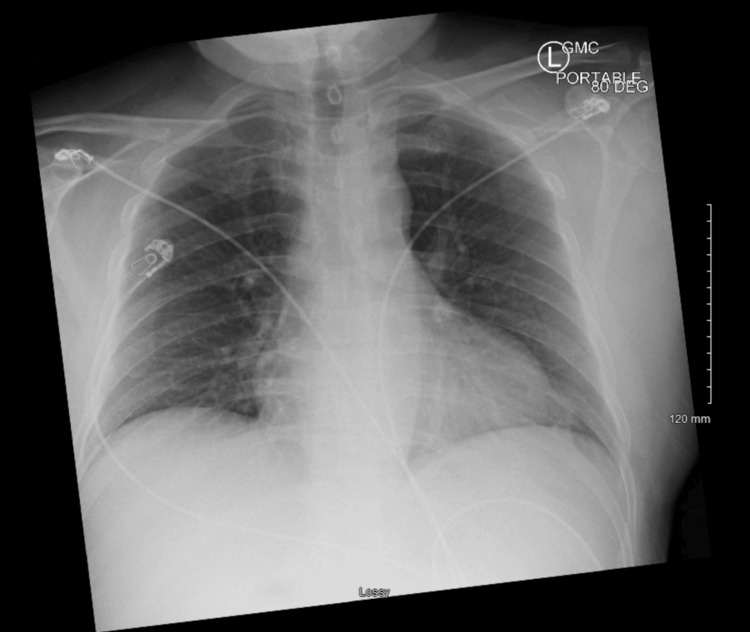
CXR in ED CXR obtained in ED showing mild diffuse interstitial opacities likely representing atelectasis secondary to low lung volumes CXR: chest x-ray; ED: emergency department

In the meantime, his initial labs resulted and were only significant for a point of care (POC) lactate level of 2.6 mmol/L and a white blood cell (WBC) count of 15.3 K/mcL with neutrophilic predominance of 90.3%. His initial high-sensitivity (HS) troponin I level was normal at 12 ng/L. Almost an hour later, he was still complaining of chest pain. A chest x-ray (CXR) was taken and was only significant for mild diffuse interstitial opacities likely representing atelectasis secondary to low lung volumes (Figure 2). He received morphine 4 mg IV and 1 more liter of lactated Ringer's. At this time, a urine drug screen had also resulted and was positive for the presence of cocaine. When asked, the patient did say that he had used cocaine the day before coming to the hospital.

**Figure 3:**
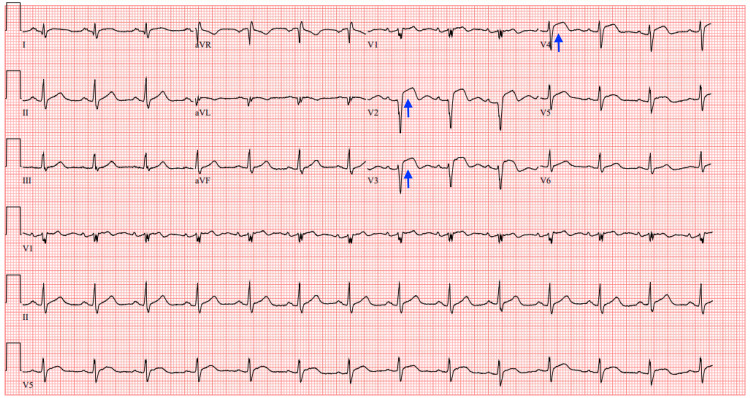
Repeat ECG after the sudden onset of chest pain Repeat ECG acquired urgently after the sudden onset of chest pain showing normal sinus rhythm at a heart rate of 82 bpm, but now with significant ST segment elevations in leads V2-V4 (blue arrows) suggesting acute anterior myocardial infarction in the territory of the left anterior descending artery ECG: electrocardiogram

Two hours after that, he started complaining of difficulty breathing, a sensation of his throat closing up, and generalized pruritus. Upon physical examination, he was noted to be diaphoretic and tachypneic. Diphenhydramine 50 mg IV was given in addition to epinephrine 0.3 mg IM. A repeat HS troponin resulted at 64 ng/L. The internal medicine service was consulted for admission, and the patient was admitted to the medical floor with no large changes to management. Six hours later, a serial HS troponin test, which had been ordered, resulted in 51,452 ng/L. The primary team was notified of this change, and an urgent ECG was obtained, which showed normal sinus rhythm at a rate of 82 bpm, but now with significant ST segment elevations in leads V2-V4 (Figure 3).

The on-call cardiology team was urgently contacted, and a stat transthoracic echocardiogram (TTE) was performed at the bedside, which showed apical anterior and inferior apical regional wall motion abnormalities consistent with a left anterior descending (LAD) coronary artery infarct in addition to moderate left ventricular hypertrophy (LVH) with a left ventricular ejection fraction (LVEF) of 40%-45% (Video 1).

**Video 1:** Initial transthoracic echocardiogram (TTE) Video of the initial obtained TTE performed at bedside showing apical anterior and inferior apical regional wall motion abnormalities consistent with a left anterior descending (LAD) coronary artery infarct in addition to moderate left ventricular hypertrophy (LVH) with a left ventricular ejection fraction (LVEF) of 40%-45%

**Figure 4:**
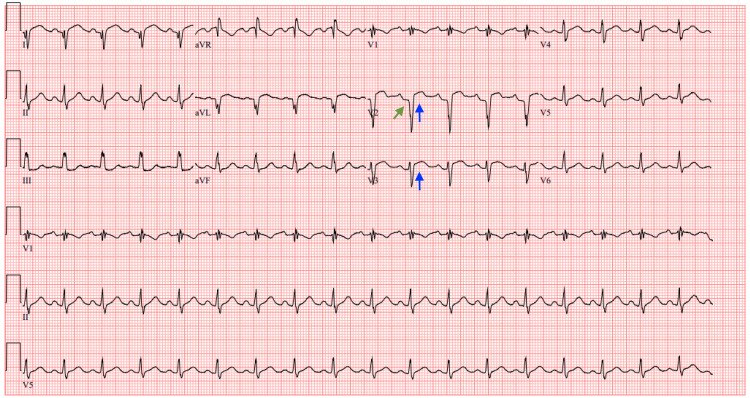
Repeat ECG obtained after TTE Repeat ECG obtained after TTE showing sinus tachycardia at a heart rate of 108 bpm, persistent ST segment elevations in leads V2-V3 (blue arrows), a Q wave in lead V2 (green arrow), and right axis deviation ECG: electrocardiogram; TTE: transthoracic echocardiogram

An urgent diagnostic coronary angiogram was recommended by the cardiology team; however, the patient declined as he was asymptomatic at that time and opted to proceed with medical management instead. Loading doses of aspirin 324 mg p.o. and clopidogrel 300 mg p.o. were given, and a heparin infusion was started in addition to lorazepam 2 mg IV. The use of β-blockers at this time was not recommended due to the recent cocaine use. A repeat ECG at this time showed sinus tachycardia at a HR of 108 bpm, persistent ST segment elevations in leads V2-V3, a Q wave in lead V2, and right axis deviation (Figure 4).

**Figure 5:**
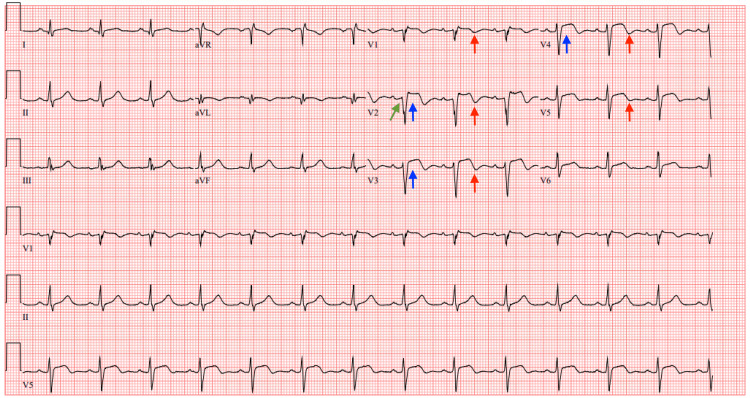
ECG on day 2 of hospital admission ECG obtained in the ICU on day 2 of hospital admission showing normal sinus rhythm at a heart rate of 82 bpm and persistent ST segment elevations in leads V2-V4 (blue arrows), with a Q wave in lead V2 (green arrow) and T wave inversions in leads V1-V5 (red arrows) ECG: electrocardiogram; ICU: intensive care unit

Considering there was a concern for type 1 KS due to an allergic reaction that is complicated by possible coronary artery vasospasm due to cocaine use and later epinephrine administration in the absence of previously known CAD, a nitroglycerin infusion was started, and the patient was transferred to the intensive care unit (ICU). Six hours later, the HS troponin level peaked at 100,818 ng/L and then started to decline afterward over multiple serial checks overnight. The following morning, another ECG was obtained, which showed normal sinus rhythm at a HR of 82 bpm and persistent ST elevations in leads V2-V4, with a Q wave in lead V2 and T wave inversions in leads V1-V5 (Figure 5).

**Figure 6:**
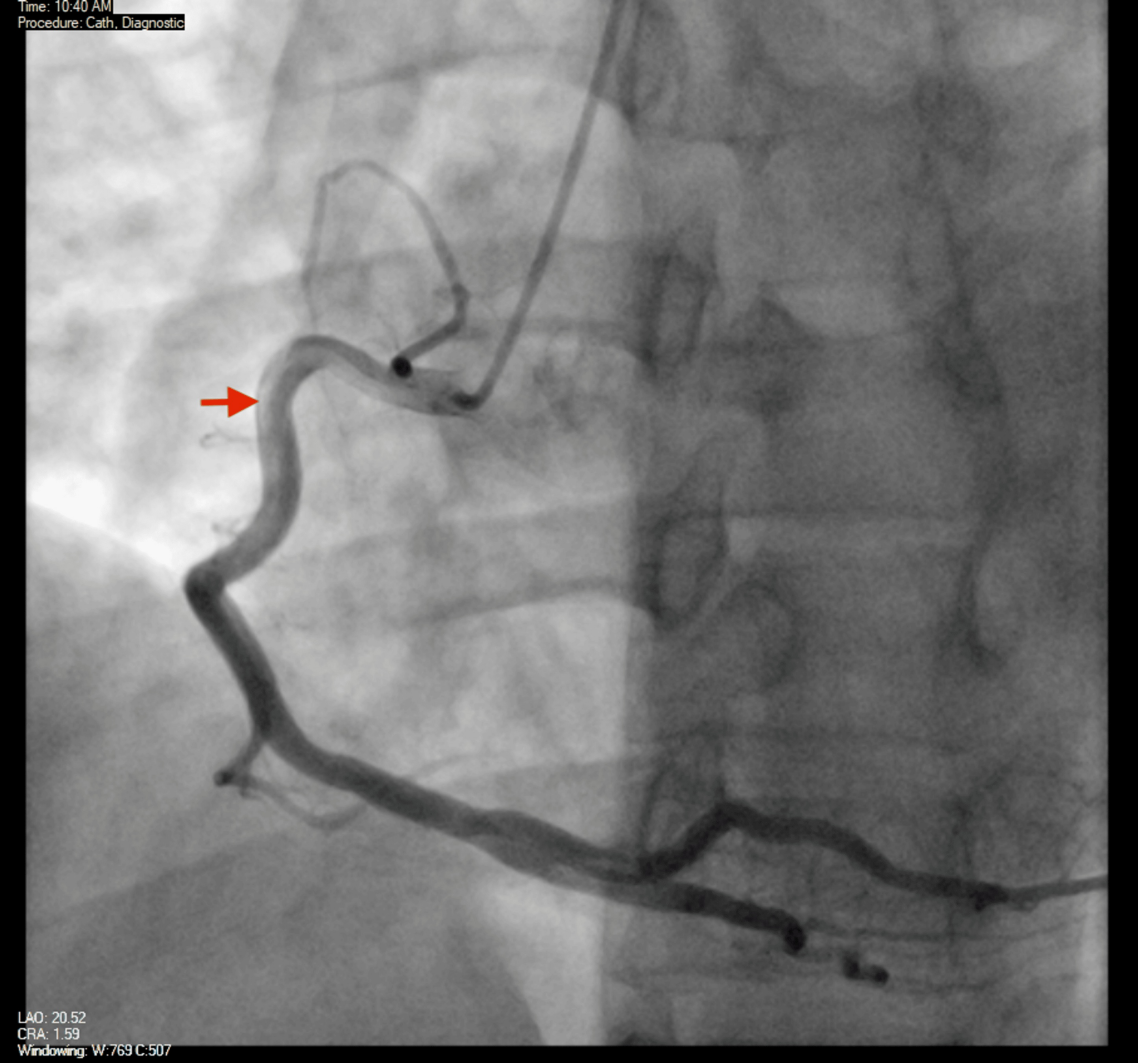
Coronary angiogram - right coronary artery Image from the coronary angiogram showing contrast filling the right coronary artery (red arrow) with no obstructive lesions

**Figure 7:**
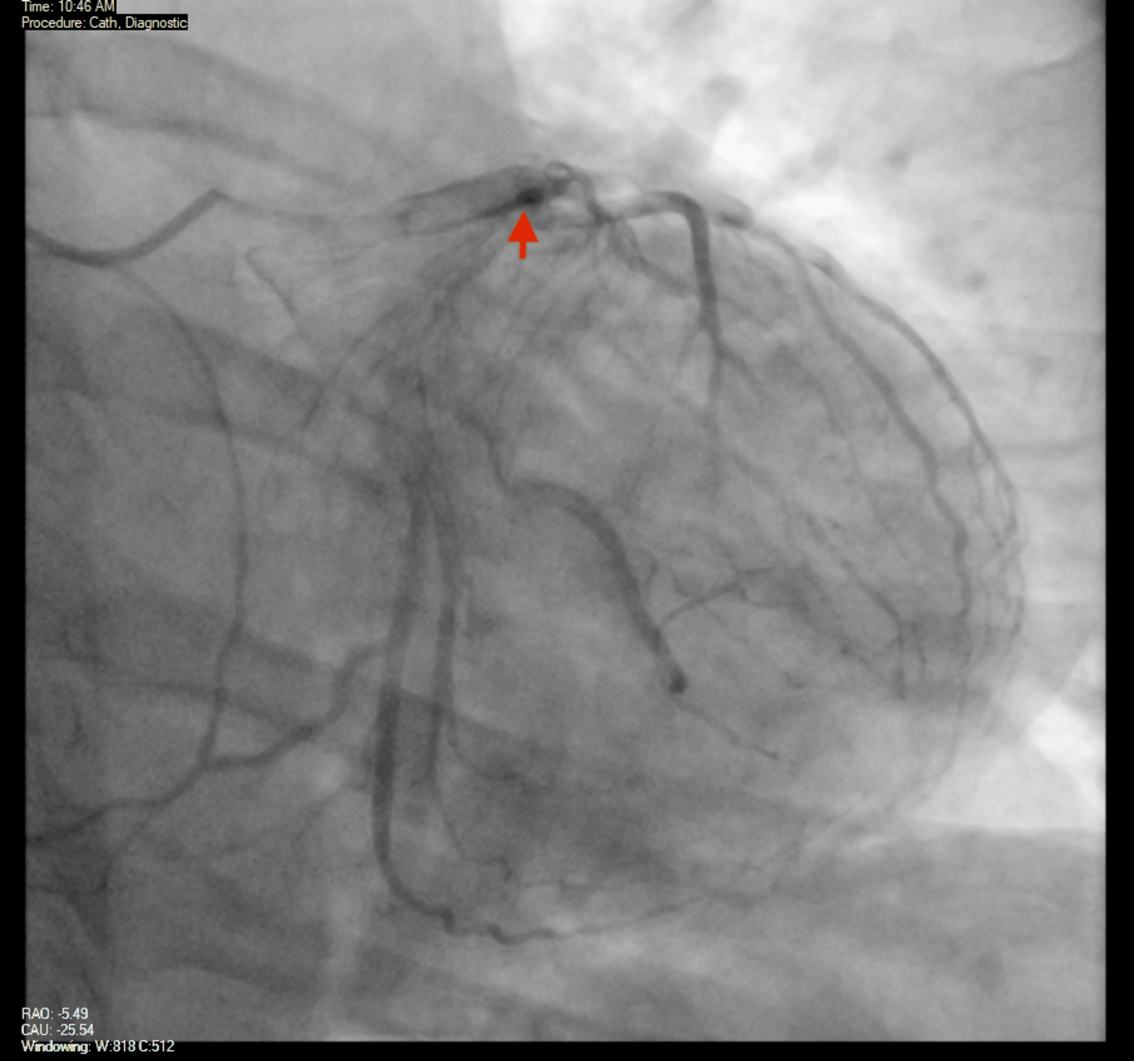
Coronary angiogram - left anterior descending (LAD) artery Image from the coronary angiogram showing a large LAD artery with proximal haziness (red arrow) that was thought to be either a thrombus or a dissection

The cardiology team discussed again with the patient the need for a diagnostic coronary angiogram, and the patient agreed to this plan. The patient subsequently underwent a coronary angiogram that noted right coronary artery dominance, with a large caliber left main coronary artery with no obstructive disease and a large LAD with proximal haziness that was thought to be either a thrombus or a dissection, in addition to TIMI I flow in the distal LAD with no flow in the apical LAD. The left circumflex coronary artery and the right coronary artery were large caliber vessels with no obstructive disease as well. This was interpreted as a likely acute plaque rupture in the proximal LAD with a layered thrombus and distal embolization (Figures 6, 7).

No percutaneous coronary angiography (PCI) was done at the time due to concerns about drug abuse and medication compliance. The cardiology team recommended the continuation of IV heparin infusion for a total of 48 hours, in addition to the continuation of the nitroglycerin infusion, aspirin 81 mg p.o. daily, and clopidogrel 75 mg p.o. daily, adding amlodipine 2.5 mg p.o. daily. It was also recommended to start an infusion of eptifibatide for a total duration of 18 hours.

**Figure 8:**
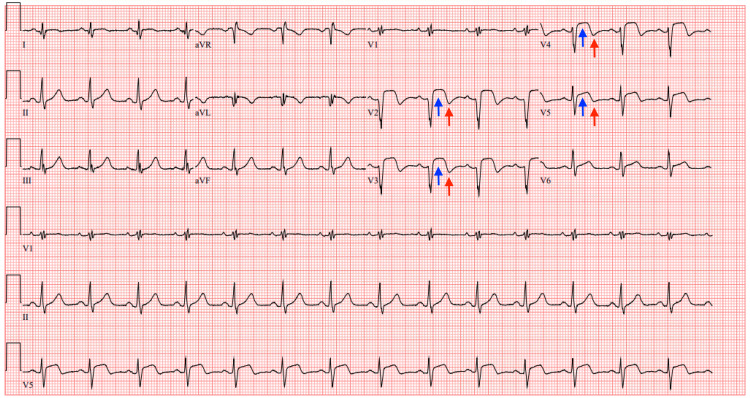
ECG on day 3 of hospital admission ECG obtained in the ICU on day 3 of hospital admission showing normal sinus rhythm at a heart rate of 86 bpm, in addition to persistent ST segment elevations in leads V2-V5 (blue arrows) with T wave inversions in leads V2-V5 (red arrows) ECG: electrocardiogram; ICU: intensive care unit

On day three of hospital admission, he remained free of chest pain, and the eptifibatide and nitroglycerin infusions expired. An ECG obtained showed normal sinus rhythm with a HR of 86 bpm, in addition to persistent ST segment elevations in leads V2-V5 with T wave inversions in leads V2-V5 (Figure 8).

**Figure 9:**
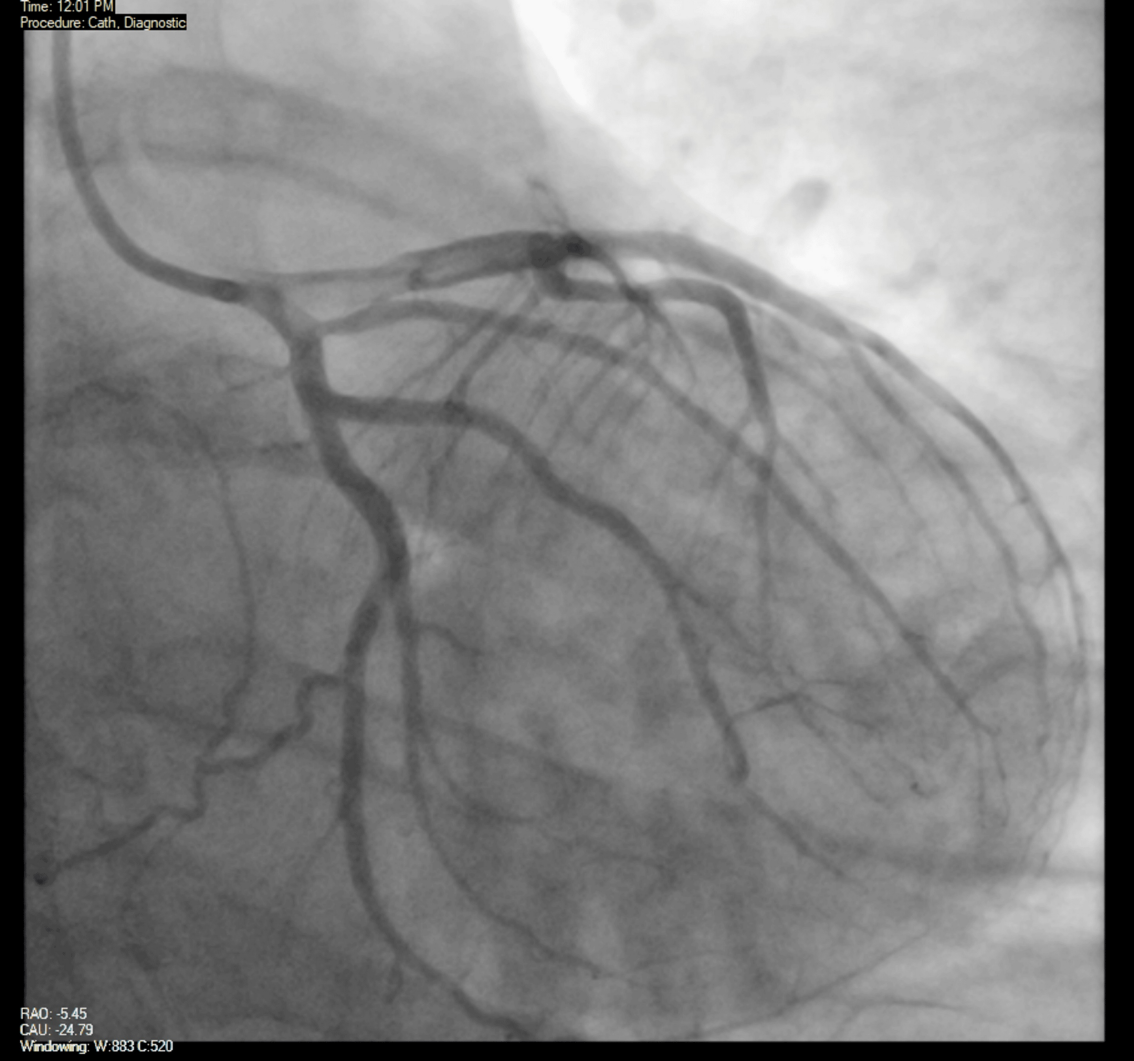
Repeat coronary angiogram Image from the repeat coronary angiogram showing large caliber coronary vessels with no obstructive lesions. It is noted that the previously visualized haziness in the proximal LAD artery is no longer present LAD: left anterior descending

The cardiology team offered to perform PCI; the patient initially declined but then agreed to the procedure. On day four of hospital admission, the patient underwent a second coronary angiography that showed significant improvement of the haziness that was previously noted in the proximal LAD (Figure 9). On review of the IVUS, no evidence of plaque rupture or plaque erosion was seen (Video 2), and what appears to be an organized clot was visualized in the LAD. This was thought to be more consistent with type 1 KS as opposed to plaque rupture or coronary dissection. The distal embolization had resolved with significant improvement in blood flow. Given these findings, the cardiology team recommended continuing with medical therapy instead of performing PCI. This was consistent with the previous diagnosis of KS, considering the timeline of the condition that started after the administration of medications. It was recommended that the patient also be started on apixaban 5 mg twice daily and continue with triple therapy with aspirin, clopidogrel, and apixaban for a duration of one week, with discontinuation of aspirin afterward and maintenance of medical therapy on clopidogrel and apixaban daily. It was also recommended that the patient be started on angiotensin-converting enzyme (ACE) inhibitor or angiotensin receptor blocker (ARB) therapy.

**Video 2:** Intravenous ultrasound (IVUS) of the proximal left anterior descending (LAD) coronary artery performed during the second coronary angiography IVUS performed during the second coronary angiography, which did not reveal any evidence of plaque disruption or rupture

The patient returned to the ICU without any complaints or complications. On day five of hospital admission, a repeat TTE was performed, which showed residual hypokinesis of the apical and anterior apical segments but with improvement of LVEF to approximately 50% (Video 3).

**Video 3:** Repeat transthoracic echocardiography (TTE) Repeat TTE after treatment with heparin infusion showing residual hypokinesis of the apical and anterior apical segments but with improvement of LVEF to approximately 50% LVEF: left ventricular ejection fraction

**Figure 10:**
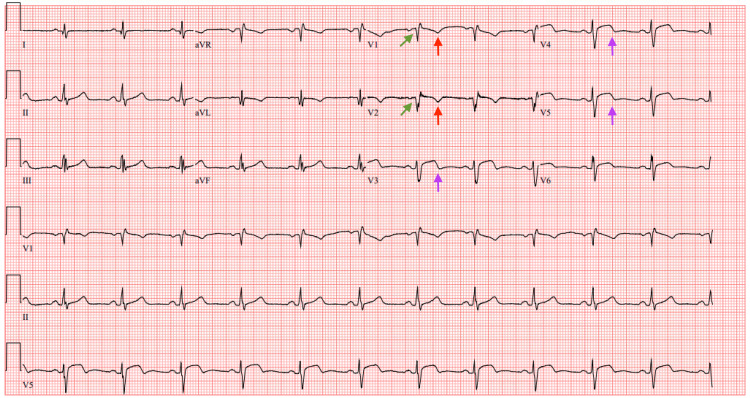
ECG on day 5 of hospital admission ECG obtained in the ICU on day 5 of hospital admission showing normal sinus rhythm at a heart rate of 71 bpm. Q waves were noted to be present in leads V1-V2 (green arrows) with persistent T wave inversions in leads V1-V2 (red arrows) and biphasic T waves in leads V3-V5 (purple arrows) ECG: electrocardiogram; ICU: intensive care unit

An ECG showed normal sinus rhythm with a HR of 71 bpm. Q waves were noted to be present in leads V1-V2 with persistent T wave inversions in leads V1-V2 and biphasic T waves in leads V3-V5 (Figure 10).

The patient was transferred from the ICU to the medical floor and was subsequently discharged home on the same day with recommendations to continue medical therapy with aspirin, clopidogrel, and apixaban as stated above, in addition to amlodipine and an ACE inhibitor/ARB. He was also counseled on cocaine use cessation multiple times during this hospital admission and immediately prior to hospital discharge.

## Discussion

KS is an entity best described as an allergic acute coronary syndrome (ACS). Its prevalence in the United States among patients hospitalized for allergic anaphylactic reactions is approximately 1.1%, with an all-cause inpatient mortality rate of 7.0% [6]. This hypersensitivity reaction usually occurs secondary to an immune system reaction against allergens such as medications, food, or other environmental factors. It is theorized that the mechanism by which this allergic reaction occurs is through the sudden activation of mast cells primarily by IgE-mediated allergic reactions in addition to other pathways including activated complement proteins or other stimuli. This will eventually lead to the release of stored inflammatory mediators, which in turn will promote histamine release that results in plaque disruption by means of increasing hemodynamic stress and precipitating coronary arterial vasospasm [7]. Some of the most commonly implicated medications include analgesics such as nonsteroidal anti-inflammatory drugs (NSAIDs), anesthetic agents, antimicrobials including penicillins and cephalosporins, antineoplastic medications, contrast media, proton pump inhibitors, and often, ironically, medications usually used to treat allergic reactions including steroids and adrenergic agonists. Other conditions that may precipitate KS include those related to allergic manifestations such as angioedema, bronchial asthma, and anaphylaxis [8]. Three types of KS have been reported: type 1, which occurs in patients without CAD or cardiovascular risk factors and is thought to be mediated by coronary vasospasm; type 2, which occurs in patients with pre-existing CAD and is thought to be related to plaque erosion or rupture; and type 3, which occurs in patients with coronary artery stents and is characterized by chronic inflammation after the implantation of said stent [7].

In terms of diagnosis and management, no specific guidelines exist, and in these cases, the rationale for management resembles that for ACS in general [9]. The first step is to maintain a high index of suspicion especially when an allergic reaction is reported by the patient such as in this case. Otherwise, further means of investigation might well be through laboratory testing for allergic markers and cardiac enzymes and obtaining an ECG to assess for ischemic changes and an echocardiogram to assess for signs of myocardial damage such as wall motion abnormalities, in addition to coronary angiography and IVUS, which would determine whether previous obstructive CAD exists or whether there is any plaque rupture or erosion, thus contributing to the classification of KS and further management [8]. As for what relates to the management, it is often focused on the management of the initial allergic reaction to prevent further potential immune response and subsequent myocardial damage in addition to the management of the ACS itself. While no dedicated guidelines for KS itself exist, consensus-based approaches and case-based strategies are often utilized. It is often suggested that the use of antihistamines might be helpful in the management of mild to moderate allergic manifestations and an adrenergic agonist agent such as epinephrine in severe cases like anaphylaxis. It is also suggested that glucocorticoids might be helpful to alleviate the immune reaction and help prevent its progression but may not be effective in the emergent setting [7,8,10]. Other medications that might also be beneficial in controlling the allergic reaction are mast cell stabilizers since they directly affect mast cell activation, which is the proposed main mechanism behind this syndrome [10,11]. In terms of the management of the ACS component of KS, the general guidelines for the management of ACS are used. Management starts with agents for reversal of vasospasm including nitroglycerin and calcium channel blockers. This can be useful in cases of type I KS. Loading with antiplatelet agents such as aspirin and clopidogrel is reasonable in cases of type 2 KS in addition to an anticoagulation agent such as heparin. The use of β-blockers is generally not recommended, as any β-blockade effect may lead to an unopposed α-adrenergic agonist effect, which would potentially result in coronary vasospasm. It is suggested that opioids be used with caution as they might induce mast cell activation, thus leading to the worsening of the condition. Cases of type 3 KS might also benefit from loading with antiplatelet agents and anticoagulation such as in type 2 KS, in addition to coronary angiography for possible aspiration of intracoronary thrombosis [10,11].

In cases of KS, such as in this case specifically, we believe that urgent evaluation by the cardiology team is of the highest importance, not only for guidance on investigation but also for management, ultimately. For our patient, considering the confounding factor of recent cocaine use, it was difficult to define a single reason behind this presentation; however, since the patient's initial troponin levels were normal and only started rising after the administration of epinephrine for anaphylaxis, his condition was treated as KS instead of cocaine-induced vasospasm, although both conditions could have overlapped. Management was partly driven by the unique situation that was at hand. The patient initially refused to undergo coronary angiography, so medical management was started by means of heparin infusion for ACS and nitroglycerin infusion to promote vasodilation. However, he then underwent angiography, which did not show significant obstructive CAD, so he did not receive PCI. Management of the coronary thrombus was by means of oral anticoagulation due to concerns for further clot formation. We also believe that continued follow-up with cardiology in the outpatient setting is imperative to the recovery journey.

## Conclusions

KS is a rare but critical cause of ACS triggered by allergic reactions. Timely recognition and management are of utmost importance and might slightly differ based on the subtype, which is usually designated by the absence or presence of CAD or coronary stents. This case was riveting because of multiple possible instigators, interdisciplinary team discussions, and the utilization of IVUS to aid in diagnosis and management. Current treatment is based on general ACS management guidelines; however, different approaches may be considered based on syndrome subtype, most notably where it relates to β-blockers, as they are a staple of management for ACS but might be counterproductive if they lead to unopposed α-adrenergic agonist effect potentially worsening vasospasm. Type 1 KS might benefit from administration agents to reverse coronary vasospasm, type 2 KS might benefit from anticoagulation as well, considering the existence of CAD, and type 3 KS might benefit from coronary angiography and thrombectomy when present. Increased awareness and further research are needed to improve diagnosis and treatment outcomes.
